# Surgery for Cystadenoma of the Retromolar Pad Area With Reconstruction Using a Buccal Fat Pad Flap: A Case Report

**DOI:** 10.7759/cureus.27314

**Published:** 2022-07-26

**Authors:** Yukio Watabe, Shota Shinagawa, Shiro Shigematsu

**Affiliations:** 1 Department of Dentistry and Oral Surgery, Tokyo Metropolitan Tama Medical Center, Fuchu-shi, JPN

**Keywords:** retromolar pad area, reconstructive surgery, salivary gland tumor, cystadenoma, buccal fat pad flap

## Abstract

Salivary gland tumors can also occur rarely in the retromolar area, though common near the junction of hard and soft palate, labial mucosa, and buccal mucosa. Most salivary gland tumors in the retromolar pad area are malignant and should be excised. The cystadenoma is a rare, benign, salivary gland tumor. Importantly, incomplete resection of this tumor can lead to recurrence or cervical lymph node metastasis. We reported herein a case of cystadenoma arising in the right retromolar pad area in a 63-year-old male patient who underwent reconstruction using a buccal fat pad flap (BFPF) after the surgical removal of the tumor with a 10-mm margin left a defect with bone exposure. No evidence of recurrence or complication was found at the postoperative, three-year follow-up.

## Introduction

Salivary gland tumors are relatively rare in the oral cavity but can potentially affect the minor salivary glands significantly. Minor salivary gland tumors comprise fewer than 25% of salivary gland tumors [1]. The cystadenoma is a rare, benign, salivary gland tumor and accounts for 4% of salivary gland neoplasms [2]. It commonly arises on the hard palate, buccal mucosa, or rarely in the retromolar pad area [3-5]. The recommended treatment for a cystadenoma is complete surgical resection [6], but reconstruction of the retromolar pad area is critical to maintaining the function of the oral cavity.

The buccal fat pad flap (BFPF) is a reliable and straightforward method of treating intraoral soft tissue defects. It has been used to reconstruct maxillary defects induced by tumors since it was first reported in 1977 [7]. We herein describe a surgically resected cystadenoma for which reconstruction using a BFPF was performed to correct a defect with bone exposure left by the resection.

## Case presentation

**Figure 1:**
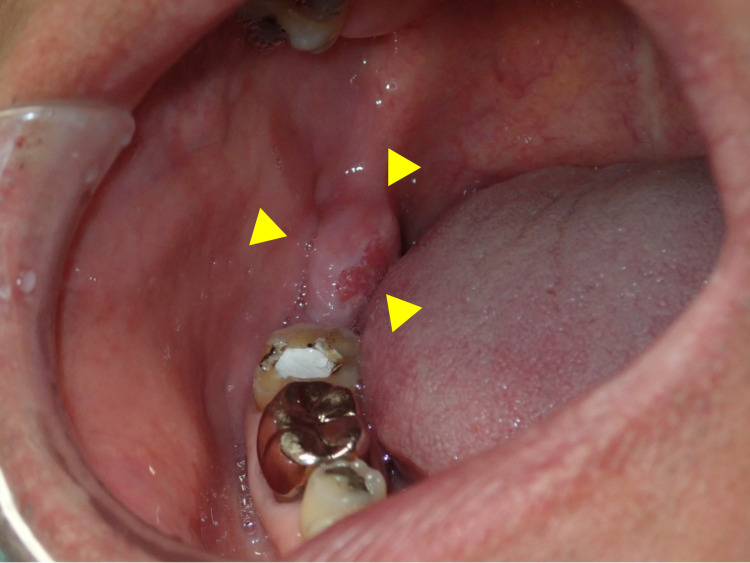
Intraoral photograph showing the tumor in the retromolar pad area

A 63-year-old male patient was referred by his dentist to our hospital to treat an asymptomatic mass in the right retromolar pad area, which was discovered incidentally during dental treatment. There was no history of oral surgery and oral trauma. The patient had no history of smoking or alcohol consumption and no exposure to environmental carcinogens. Clinical examination revealed a mass of 17 mm x 10 mm without tenderness and a reddish area with discrete papules bordered by a peripheral whitish area on the lingual aspect in the right retromolar pad area (Figure 1).

**Figure 2:**
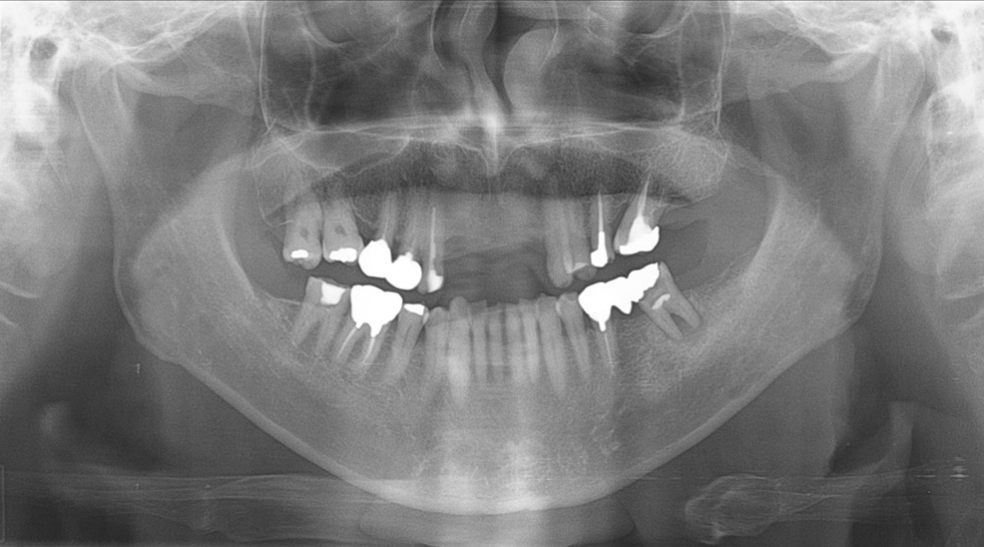
Orthopantomograph showing the right mandible without any bony invasion of the tumor

The patient’s mouth opening was unconstrained. The patient had no difficulties in swallowing and eating. The patient had a history of hyperuricemia. Orthopantomography revealed no erosive destruction or changes in the trabecular bone marrow pattern in mandibular alveolar bone and no presence of radiolucency beneath the radiopaque-filling material involving the underlying pulp with an ill-defined radiolucency with loss of lamina dura in periapical root region of 19 (International Tooth Numbering System), suggestive of secondary caries with a chronic periapical abscess in the mandible (Figure 2).

**Figure 3:**
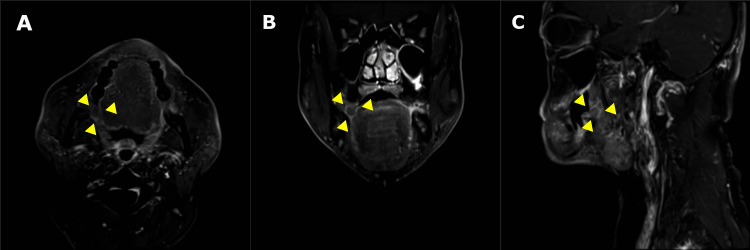
Short TI inversion recovery magnetic resonance image with contrast showing the lesion in the right retromolar area. (A) Axial plane, (B) coronal plane, and (C) sagittal plane.

Contrast-enhanced magnetic resonance imaging (MRI) with short tau (TI) inversion recovery demonstrated a hypointense lesion with a sharp margin, round shape, and uniform density distribution lesion in the right retromolar area, which was 13 mm x 10 mm in size and had not invaded the surrounding tissue (Figure 3).

Squamous cell carcinoma or malignant salivary gland tumor was provisionally diagnosed. An incisional biopsy was performed, and a sialadenoma papilliferum was diagnosed. However, since salivary gland tumors in the retromolar pad area are more frequently malignant tumors, total excision under general anesthesia was planned. The procedure included a wide tumor excision with a 10-mm margin. Because of its proximity to 47, the tooth was extracted. The inferior alveolar nerve was preserved during mass resection.

**Figure 4:**
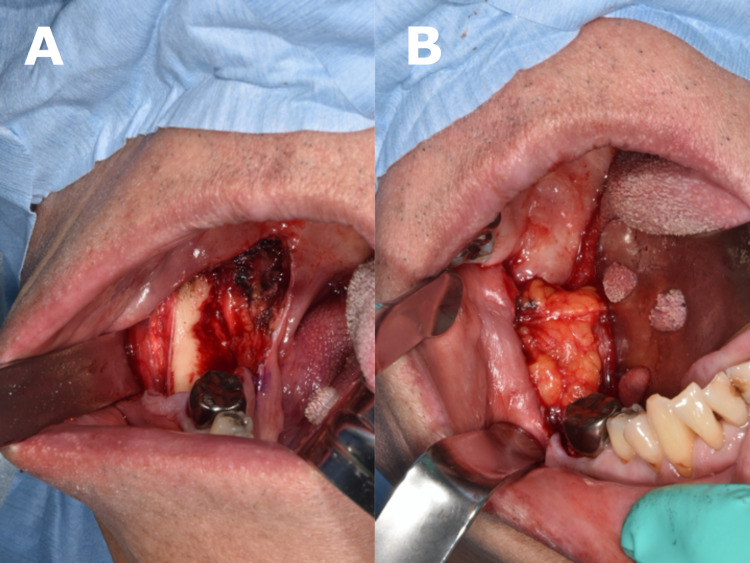
(A) Surgical site after the tumor resection. (B) Buccal fat pad exposure in the oral defect.

With mandibular bone exposure, the resection resulted in a sizable oral defect in the retromolar pad area. The oral defect was reconstructed using a BFPF (Figure 4, Panel A). Submucosa from the resection margin of the buccal mucosa was peeled and elevated cranially. The buccal muscle was incised cranially at the pterygomandibular ligament, and the buccal fat pad (BFP) was identified anterior to the medial pterygoid muscle. Blunt dissection was done, and the BFP pulled out anteriorly toward the oral defect without tension. The flap margin was sutured and fixed to the marginal oral mucosa with 4-0 nylon. The oral defect in the retromolar pad area was covered entirely with the BFP (Figure 4, Panel B). No additional skin grafting was performed because BFP flaps epithelialize spontaneously.

**Figure 5:**
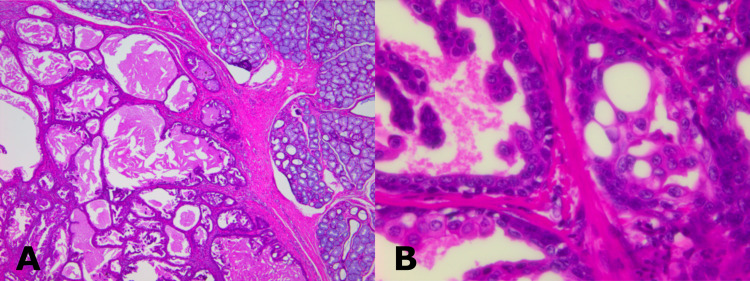
(A) Histological sections showing multiple cystic spaces of variable sizes with central eosinophilic material (hematoxylin and eosin stain, 40x). (B) Cystic cavity lined by bilayered epithelium consisting of columnar and flat epithelium exhibiting intraluminal papillary projections (hematoxylin and eosin stain, 400x).

A thorough examination of the excised specimen led to the diagnosis of a cystadenoma (Figure 5, Panels A and B).

**Figure 6:**
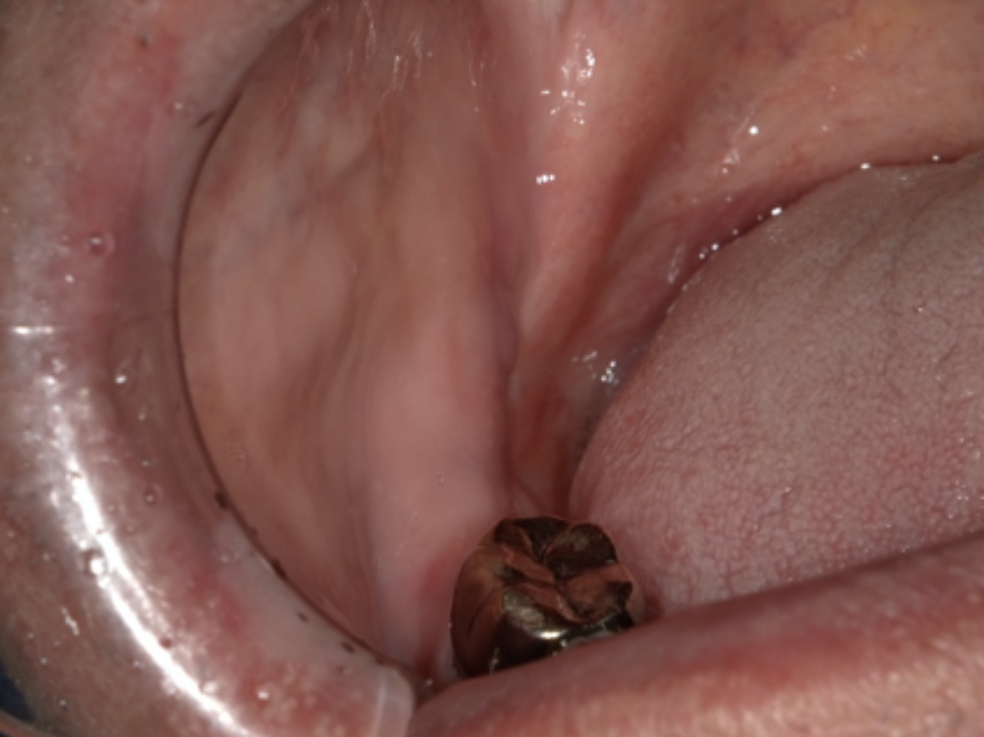
Intraoral photograph showing no scar contracture or recurrence at postoperative year 3

Surgical antibiotic prophylaxis (ampicillin 3.0 g/day) was administered until the day after surgery. The patient began a dysphagia soft diet the day after surgery. Mouth opening exercises were started in the early postoperative period. The postoperative course was uneventful, and the patient’s mouth opening was unconstrained. No recurrence was found at the three-year follow-up (Figure 6).

## Discussion

We reported a rare case of cystadenoma in the retromolar pad area. It should be borne in mind that a neoplastic lesion developing in the retromolar pad area may be a malignant salivary gland tumor. Therefore, in the present case, we performed a wide resection and reconstructed the surgical defect using a BFPF with good outcomes.

Cystadenomas, which arise in the pancreas, ovary, prostate, or kidney [8-11], rarely occur in the retromolar pad area. When it occurs in the head and neck area, it is often found in the parotid gland, with a frequency of 45% [12]. The most frequent sites of cystadenoma development in the oral cavity are the hard palate, the buccal mucosa, and the tongue [13], and rarely, the retromolar pad area [3-5]. Table 1 summarizes the case reports of cystadenoma in the retromolar pad area published in English since 2000.

**Table 1:** The reported case of cystadenoma arising in the retromolar pad area

Case	Authors (Year)	Age	Gender	Size (mm)	Symptom	Preoperative diagnosis	Treatment	Recurrence
1	Takahashi et al. (2008) [3]	43	Male	20 × 27	Swelling	Monomorphic adenoma	Excision	No
2	Sheetal et al. (2016) [4]	40	Female	30 × 40	Swelling	Pleomorphic adenoma	Excision	No
3	Wu et al. (2018) [5]	45	Female	None stated	Recurrent painless mass in the retromolar and submandibular regions	Cystadenoma	Excision	No
4	Present case	63	Male	13 × 10	Swelling	Sialadenoma papilliferum	Excision	No

In general, it was predominantly found in females [2]. Table 1 indicated no gender difference in the case of cystadenomas in the retromolar pad area. Some reports indicated no gender difference in the frequency of lesions [14]. Standard features of benign salivary gland tumors, including cystadenoma on CT and MRI, are sharp margins, round shape, and uniform density distribution [15]. In this case, findings characteristic of a benign salivary gland tumor were observed on MRI. However, most salivary gland tumors in the retromolar pad area are malignant [16], and a wide resection under general anesthesia was ultimately performed.

The wide excision in our patient left a defect with mandibular bone exposure in the retromolar pad area, which was corrected using a BFPF. The BFPF has the advantage of being simple and easy to perform. The BFP consists of the main body with four extensions: the temporal, buccal, pterygoid, and pterygopalatine processes [17]. Generally, the BFP can easily be accessed via a horizontal incision over the periosteum near the maxillary third molar [18]. After the resection, the BFP was easily accessed in the present case by dissecting and elevating the buccal mucosa and incising the exposed buccal muscle in the surgical field. The failure rate of reconstructive procedures for oral defects using BFPF is low, thanks to the tissue's rich vascularization and rapid epithelialization [19].

According to a review of 12 studies, 89.1% of BFPF reconstructions had no associated adverse events, including infection, loss of graft, bleeding, fistula, dehiscence, and mouth opening limitation [20]. However, the BFPF has several shortcomings. First, the size of the defect that can be corrected with this procedure is less than 5 cm in diameter [21]. Importantly, constriction of mouth opening is a postoperative complication associated with reconstructive surgery using a BFPF. In a retrospective study, five of 32 patients with reconstruction using a BFPF for an oral defect after tumor resection had scar retraction and constriction of mouth opening. After reconstructive oral surgery using a BFPF, mouth opening training should be started on postoperative day 5 to avoid these complications [22].

In this case, the biopsy result was sialadenoma papilliferum. However, the resected specimen was finally diagnosed as a cystadenoma. Sialadenoma papilliferum usually occurred at the junction of the hard and soft palates and the floor of the mouth and histopathologically did not show multiple cystic cavities [23]. The histopathologic differential diagnosis of cystadenoma includes Warthin's tumor and cystadenocarcinoma. Warthin's tumor is characterized by subepithelial aggregates of lymphocytes and germinal centers. These characteristics were not observed in this case. Furthermore, there was no evidence of invasive growth in the surrounding area, and findings suggestive of cystadenocarcinoma were lacking.

Most salivary gland tumors in the retromolar pad area are malignant [16]. Although the present case was a cystadenoma, an accurate diagnosis of the minor salivary gland is often difficult, even if the biopsy results indicate a benign tumor. For this reason, even a minor salivary gland tumor in the retromolar pad area should be excised. Resection is recommended in this disease, and recurrence or malignancy is rare [24]. However, a previous study reported a case of cystadenoma recurrence in the retromolar pad at postoperative year 5 with a simultaneous cervical metastasis [5]. Although cystadenomas are a benign form of salivary adenoma, providing a sufficiently wide surgical margin is warranted, as in the present case.

## Conclusions

We experienced a case of cystadenoma occurring in the retromolar pad area, which was widely resected. The surgical defect was reconstructed using a BFPF. Most salivary gland tumors in the retromolar pad area are malignant, but a benign tumor may occasionally occur, as in the present case. Nonetheless, even a benign salivary gland tumor, especially a cystadenoma, should be excised completely to prevent a recurrence.
